# Supplementary material for: Important features of bench press performance in non-disabled and Para athletes: A scoping review
Source: PLoS One. 2024 Nov 11;19(11):e0310127. doi: 10.1371/journal.pone.0310127 (PMC11554178; doi:10.1371/journal.pone.0310127)
Supplement: S1 File — The systematic search strategy used to search scientific databases. (DOCX) [file pone.0310127.s001.docx]

**Supplement 1.** Search strings.

***PubMed strategy***

(((((((resistance training[MeSH Terms]) OR (muscle strength[MeSH Terms])) OR (weight lifting[MeSH Terms])) OR ((sports for persons with disabilities[MeSH Terms]) OR (spinal cord injury[MeSH Terms]))) OR (((((para sport[Title/Abstract]) OR (para athletes[Title/Abstract])) OR (paralympic[Title/Abstract])) OR (para powerlifting[Title/Abstract])) OR (disabled powerlifting[Title/Abstract]))) AND ((((bench press[Title/Abstract]) OR (chest press[Title/Abstract])) OR (flat bench press[Title/Abstract])) OR (powerlifting[Title/Abstract]))) AND ((((((((performance[Title/Abstract]) OR (one rep max*[Title/Abstract])) OR (one repetition max*[Title/Abstract])) OR (strength[Title/Abstract])) OR (power[Title/Abstract])) OR (kinematics[Title/Abstract])) OR (technique[Title/Abstract])) OR (maximal strength[Title/Abstract]))) NOT ((((creatine[Title/Abstract]) OR (caffeine[Title/Abstract])) OR (ergogenic[Title/Abstract])) OR (supp*[Title/Abstract]))

***SPORTDiscus strategy***

((DE "POWERLIFTING") OR (DE "WEIGHT lifting") OR (DE "RESISTANCE training") OR (DE "SPORTS training for people with disabilities") OR (AB para sport OR para athletes OR paralympic OR para powerlifting OR disabled powerlifting)) AND (AB bench press OR chest press OR flat bench press OR powerlifting) AND (AB performance OR one rep max* OR strength OR power OR kinematics OR technique OR maximal strength) NOT (AB creatine OR caffeine OR ergogenic OR supp*)

***EMBASE strategy***

('disabled sport'/exp OR 'resistance training'/exp OR 'weight lifting'/exp OR 'spinal cord injury'/exp OR 'para sport' OR 'para athletes' OR paralympic OR 'para powerlifting' OR 'disabled powerlifting') AND ('bench press' OR 'chest press' OR 'flat bench press' OR powerlifting) AND (performance OR strength OR 'one rep* max' OR power OR kinematics OR technique OR 'maximal strength') NOT (creatine:ab,ti OR caffeine:ab,ti OR ergogenic:ab,ti OR supp*:ab,ti)
