# Supplementary material for: Important features of bench press performance in non-disabled and Para athletes: A scoping review
Source: PLoS One. 2024 Nov 11;19(11):e0310127. doi: 10.1371/journal.pone.0310127 (PMC11554178; doi:10.1371/journal.pone.0310127)
Supplement: Attachment — Submitted filename: PONE.docx [file pone.0310127.s003.docx]

**Manuscript Review: PONE-D-24-20584**

**Manuscript Summary**:

The study analyzes factors influencing bench press performance in non-disabled athletes and Para athletes, aiming to provide insights for talent identification and transfer models in weightlifting. The hypothesis posits significant differences in bench press performance determinants between these two groups, influenced by variables such as age, sex, body mass, height, and muscular strength.

**Main Findings and Highlights**:

- Key determinants of bench press performance in non-disabled athletes include age, sex, body mass, height, and muscular strength.

- For Para athletes, adaptations due to disability, such as balance, stability, and modified technique, are crucial.

- The study discusses the implications for personalized talent identification and training programs in weightlifting for different athlete groups.

**Points to Consider**:

1) Grammatical or Agreement Errors:

**Consistency in Verb Tenses**: Review the entire manuscript to ensure consistency in the use of verb tenses.

**Subject-Verb Agreement**: Ensure proper agreement between subjects and verbs. For instance, on page 40, paragraph 1, line 1, the sentence could be reviewed for consistency.

2) **Contrasting Content**:

**Clarity**: Some sections might present contradictory or confusing information. A thorough review and clarification of these points are recommended.

3) **Inconsistent Results**:

**Justification of Discrepancies**: Some results appear inconsistent with the initial hypotheses or existing literature. These discrepancies should be clearly reviewed and justified within the manuscript.

4) **Argumentative and Scientific Rhetoric Improvements**:

**Strengthen Arguments**: Improve the connection between results and conclusions to ensure a solid and consistent argument.

**Technical Language**: Use more precise and technical language when describing methods and results to avoid ambiguities.

5) **Reader Engagement**:

**Practical Examples**: Incorporate practical examples or case studies to illustrate the applicability of the results in sports practice, making the manuscript more attractive to readers interested in bench press performance.

**Hypothesis Confirmation**:

Based on the main conclusions, the initial hypothesis does not appear objectively (I suggest it appears, it makes everything clearer!), however, implicitly the implied hypothesis seems partially confirmed. Significant differences were found in bench press performance determinants between non-disabled athletes and Para athletes, particularly regarding age, sex, body mass, height, and muscular strength. However, the study identified areas where results did not fully align with the hypothesis, such as the lack of comprehensive studies across multiple domains and the underrepresentation of female participants.

**Conclusion Review**:

The conclusion partially addresses the study's objectives. It provides valuable insights into bench press performance characteristics but could more explicitly link results to the study's initial objectives. Emphasizing how the findings contribute to understanding performance determinants and informing talent identification and transfer models in weightlifting would enhance the conclusion.

**Recommendations**:

**Grammar and Agreement**: Conduct a thorough grammatical review, focusing on verb tense consistency and subject-verb agreement.

**Clarify Content**: Address and resolve any contradictory information to ensure clarity.

**Justify Inconsistencies**: Clearly explain any discrepancies between results and initial hypotheses.

**Strengthen Arguments**: Enhance the connection between results and conclusions with precise, technical language.

**Engage Readers**: Use practical examples or case studies to illustrate the real-world applicability of the findings.

**Conclusion Enhancement**: Directly link the results to the study's objectives, providing a robust synthesis of findings.

By addressing these points, the manuscript will improve in clarity, cohesion, and impact, making it more informative and engaging for readers interested in bench press performance in non-disabled and Para athletes.
