# Supplementary material for: Important features of bench press performance in non-disabled and Para athletes: A scoping review
Source: PLoS One. 2024 Nov 11;19(11):e0310127. doi: 10.1371/journal.pone.0310127 (PMC11554178; doi:10.1371/journal.pone.0310127)
Supplement: Attachment — Submitted filename: PONE-D-24-20584v1.doc [file pone.0310127.s004.doc]

**Model for Manuscript Review**

**Research articles**

**Manuscript title:**

Important Features of Bench Press Performance in Non-Disabled and Para Athletes: A Scoping Review

| **Títle** |  |
| --- | --- |
| Is it understandable and concise? | ( x ) Yes ( ) Not |
| Reflects the content? | ( x ) Yes ( ) Not |
| **Abstract** |  |
| It includes: objectives, methodology, key findings and conclusions? | ( ) Yes ( x ) Not |
| **Introduccion** |  |
| The investigation was carried out in a suitable theoretical structure? | ( ) Yes ( x ) Not |
| Clear leaves the questions you want to answer and objectives of the work? | ( ) Yes ( x ) Not |
| The cited references are current and relevant? | ( ) Yes ( x ) Not |
| **Methods** |  |
| The methods presented are appropriate to achieve the proposed objectives? | ( ) Yes ( x ) Not |
| The selection and composition of the sample are adequately described? | ( ) Yes ( x ) Not |
| The data collection process and the tools used are described clearly? | ( x ) Yes ( ) Not |
| The statistical analysis and the research design appropriate? | ( x ) Yes ( ) Not |
| **Results** |  |
| The presentation of the results clear? | ( x ) Yes ( ) Not |
| The main results are highlighted without the inclusion of interpretation and comparisons? | ( x ) Yes ( ) Not |
| The results evaluate the proposed objectives? | ( x ) Yes ( ) Not |
| Tables and figures are properly numbered, labeled and explained? | ( x ) Yes ( ) Not |
| **Discussion and Conclusion** |  |
| The results are discussed based on the literature? | ( x ) Yes ( ) Not |
| Author's interpretations show the safety and soundness? | ( x ) Yes ( ) Not |
| The limitations of the work are presented? | ( x ) Yes ( ) Not |
| The conclusions of the study are presented? | ( x ) Yes ( ) Not |
| The conclusions respond to the objectives? | ( x ) Yes ( ) Not |

**General comments:**

**Title**

Are presented satisfactorily.

**Abstract**

It is written in a structured way, however, the methodology is written in a very summarized way which ends up making the findings and conclusions of the article.

It would be very important to have more details on how the study was carried out, such as query words, length of time it was consulted, that is, articles published within a certain period of time.

Furthermore, more numerical data should be presented in order to provide an initial overview of the manuscript.

Please confirm that the keywords are listed as descriptors in health sciences.

**Introduction**

Although the introduction is of a very good length, and initially with a contextualization of Powerlifting, whether conventional or palympic, and more specifically the bench press. However, the introduction cannot move from the general to the specific, that is, the association between performance and anthropometric characteristics associated with health. And on the other hand, the problem is not clear, not allowing us to understand the objectives, much less justify them. It would also be important to present research hypotheses to be answered.

**Methods**

It should present more clearly the design of the study.

The keywords researcher and the period of time researched are not included in the manuscript. As it turns out, the database and the language, therefore, the methodology is not complete, it would be good to complement it.

**Results**

Are presented satisfactorily.

**Discussion**

It should reaffirm the objectives and start discussing the results in the chronological order that appear in the item results.

Please present the limitations of the study.

**Conclusion**

Are presented satisfactorily.

**References**

Are presented satisfactorily, review their formatting. Of the 71 references, 37 are current and 34 have been published for more than five years. In this sense, it would be good to check current studies and, if necessary, include them in the manuscript.

**Overview**

The manuscript presented addresses a relevant research topic.

It would be advisable to do a general review.

**Specific comments and suggestions:**

**Outcome evaluation**

- Accept unchanged	(	)
- Accepted with minor changes	(	)
- Accepted with major changes	( x )
- Rejected	(	)
