# Supplementary material for: Important features of bench press performance in non-disabled and Para athletes: A scoping review
Source: PLoS One. 2024 Nov 11;19(11):e0310127. doi: 10.1371/journal.pone.0310127 (PMC11554178; doi:10.1371/journal.pone.0310127)
Supplement: Attachment — Submitted filename: ResponsesToReviewers_R1.docx [file pone.0310127.s005.docx]

**Manuscript PONE-D-24-20584, titled “Important Features of Bench Press Performance in Non-Disabled and Para Athletes: A Scoping Review”**

*We would like to thank the Reviewers for providing valuable feedback on the manuscript. Addressing the comments has improved the quality of the paper. Changes to the manuscript are outlined below and are marked in red text in the revised manuscript document.*

**Reviewer #1**
Manuscript Summary:
The study analyzes factors influencing bench press performance in non-disabled athletes and Para athletes, aiming to provide insights for talent identification and transfer models in weightlifting. The hypothesis posits significant differences in bench press performance determinants between these two groups, influenced by variables such as age, sex, body mass, height, and muscular strength.

Main Findings and Highlights:
- Key determinants of bench press performance in non-disabled athletes include age, sex, body mass, height, and muscular strength.
- For Para athletes, adaptations due to disability, such as balance, stability, and modified technique, are crucial.
- The study discusses the implications for personalized talent identification and training programs in weightlifting for different athlete groups.

Response: *Thank you for taking the time to provide comments on the manuscript.*

Points to Consider:
1) Grammatical or Agreement Errors:
Consistency in Verb Tenses: Review the entire manuscript to ensure consistency in the use of verb tenses.
Subject-Verb Agreement: Ensure proper agreement between subjects and verbs. For instance, on page 40, paragraph 1, line 1, the sentence could be reviewed for consistency.

Response: *As requested, we have reviewed the entire manuscript for consistency when using verbs. Regarding the specific comment referring to page 40, paragraph 1, line 1, we are unclear what is being referred to, as page 36 onwards contains only the reference list.*

2) Contrasting Content:
Clarity: Some sections might present contradictory or confusing information. A thorough review and clarification of these points are recommended.

Response: *It is unclear which specific section or content of the manuscript is being referred to. If specific comments to specific sections of the manuscript are provided, we are willing to make amendments to address any concerns.*

3) Inconsistent Results:
Justification of Discrepancies: Some results appear inconsistent with the initial hypotheses or existing literature. These discrepancies should be clearly reviewed and justified within the manuscript.

Response: *Thank you for this comment. As this is a scoping review, we did not make any initial hypothesis. As such, we are confused as to how the results appear to be inconsistent with the hypotheses, as none were made.*

4) Argumentative and Scientific Rhetoric Improvements:
Strengthen Arguments: Improve the connection between results and conclusions to ensure a solid and consistent argument.
Technical Language: Use more precise and technical language when describing methods and results to avoid ambiguities.

Response: *The methods section has been written based on the guidelines provided by Arksey and O’Malley (2005) for scoping reviews. We have been as precise as possible, while providing enough information to replicate the search and methods used. Our use of technical language was guided by the scoping review guidelines set out by by Arksey and O’Malley (2005).*

5) Reader Engagement:
Practical Examples: Incorporate practical examples or case studies to illustrate the applicability of the results in sports practice, making the manuscript more attractive to readers interested in bench press performance.

Response: *As this is a scoping review, we can only include studies that fit our inclusion criteria. None of the studies remaining following full text screening were case studies, as a result it would be inappropriate to include discussion of these studies.*

Hypothesis Confirmation:
Based on the main conclusions, the initial hypothesis does not appear objectively (I suggest it appears, it makes everything clearer!), however, implicitly the implied hypothesis seems partially confirmed. Significant differences were found in bench press performance determinants between non-disabled athletes and Para athletes, particularly regarding age, sex, body mass, height, and muscular strength. However, the study identified areas where results did not fully align with the hypothesis, such as the lack of comprehensive studies across multiple domains and the underrepresentation of female participants.

Response: *We have not explicitly stated a hypothesis (or hypotheses). We are not comfortable generating hypotheses after conducting the scoping review. The scoping review methods were preregistered on the Open Science Framework (*[*https://doi.org/10.17605/OSF.IO/Z52XY*](https://doi.org/10.17605/OSF.IO/Z52XY)*). In the preregistration, we have not declared any hypotheses. Further, we are also not conducting any null hypothesis significance testing. Hypothesizing after the results are known has been identified as a questionable research practice (Caldwell et al. 2020) and we hope the reviewer understands that we are not comfortable with adopting this practice. Further, the aim of a scoping review is to summarize and describe findings within a research area (Arksey and O’Malley, 2005), and hypothesizing is typically not consistent with the aims of a scoping review. As we have not generated a hypothesis, we cannot confirm the hypothesis as suggested by the reviewer.*

References:

1. *Arksey, H., & O'Malley, L. (2005). Scoping studies: towards a methodological framework. International journal of social research methodology, 8(1), 19-32.*
2. *Caldwell, A. R., Vigotsky, A. D., Tenan, M. S., Radel, R., Mellor, D. T., Kreutzer, A., ... & Boisgontier, M. P. (2020). Moving sport and exercise science forward: a call for the adoption of more transparent research practices. Sports Medicine, 50(3), 449-459.*

Conclusion Review:
The conclusion partially addresses the study's objectives. It provides valuable insights into bench press performance characteristics but could more explicitly link results to the study's initial objectives. Emphasizing how the findings contribute to understanding performance determinants and informing talent identification and transfer models in weightlifting would enhance the conclusion.

Response: *It is unclear whether any changes to the conclusion are being suggested.*

Recommendations:
Grammar and Agreement: Conduct a thorough grammatical review, focusing on verb tense consistency and subject-verb agreement.
Clarify Content: Address and resolve any contradictory information to ensure clarity.
Justify Inconsistencies: Clearly explain any discrepancies between results and initial hypotheses.
Strengthen Arguments: Enhance the connection between results and conclusions with precise, technical language.
Engage Readers: Use practical examples or case studies to illustrate the real-world applicability of the findings.
Conclusion Enhancement: Directly link the results to the study's objectives, providing a robust synthesis of findings.
By addressing these points, the manuscript will improve in clarity, cohesion, and impact, making it more informative and engaging for readers interested in bench press performance in non-disabled and Para athletes.

Response: *We thank the reviewer for the time taken to review the manuscript. We have attempted to address the reviewers concerns as best we can, reviewing the manuscript based on suggestions and making amendments where possible. However, without being provided with specific comments (or examples) or specific sections, it is difficult to understand which specific aspects of the manuscript are being referred to.*

**Reviewer #2**

This scoping review explored variables related to bench press performance (1RM) in both able-bodied and para-athlete populations. Of the 32 studies included, the authors noted body composition and anthropometric variables had the strongest association with bench press performance. The study is well-written and fills an important gap in the literature. I have minor specific comments below.

Response: *We thank the reviewer for their time taken to critique this manuscript. Addressing the concerns raised has improved the quality of the manuscript.*

1) Abstract– If character limits allow, could the authors identify all six of the domains into which features were grouped?

Response: *Domains are now included in the abstract as requested.*

2) Introduction – the introduction is concise, references relevant literature, and provides the rationale for the current study.

Response: *Thank you for this positive comment.*

3) Methods, exclusion criteria – It seems that excluding those who cannot bench press their body weight (especially for women) artificially limits the ranges of bench press performance for analysis. To me, this could limit valuable information on the predictors of performance if those with lower performance are not included. Could the authors include an analysis without this exclusion criterion (being able to bench press body mass) to see if the analysis changes?

Response: *Thank you for this important comment. This was an important consideration when planning the scoping review. Our primary reasoning for excluding participants who could not bench press their body weight was to limit the inclusion of participants with minimal bench press experience. It is likely that novices will make progress quickly and it is unlikely that the factors explaining the initial increases in performance are related to improved performance in more experienced lifters. It is common for original studies to have inclusion criteria around bench press 1RM to body mass ratio. For example, the following studies examined bench press performance and recruited participants with a bench press 1RM to body mass ratio of 1.25 (Wagner et al. 1992, Journal of Applied Biomechanics), 1.34 (Willardson et al. 2006, Journal of Strength and Conditioning Research), 1.67 (Dos Santos et al. 2020, International Journal of Sports Physiology and Performance) and 1.36 (Ratamess et al. 2006, European Journal of Applied Physiology). Further, we wish to adhere to our preregistered inclusion criteria (*[*https://doi.org/10.17605/OSF.IO/Z52XY*](https://doi.org/10.17605/OSF.IO/Z52XY)*), in which we stated that studies must include participants with a bench press 1RM to body mass ratio of greater than 1. Based on the above reasons, we have not included studies recruiting participants with a bench press 1RM to body mass ratio of less than 1. We hope the reviewer understands the need to abide by our pre-registration. We believe our criteria are reflective of the wider literature and think it is unlikely that inclusion of studies with a bench press 1RM to body mass ratio <1 would alter our findings.*

4) Methods, selection criteria – was supportive equipment (e.g. bench shirt or wrist wraps) taken into account during 1RM assessment in the studies included? Please clarify.

Response:

*We have extracted whether or not supportive equipment was used by studies during 1RM assessments (lines142 to 145):*

*“We also extracted whether or not supportive equipment (e.g. bench shirt or wrist straps) was used during 1RM attempts. Where studies did not state that supportive equipment was used, we assumed it was not. Additionally, we assumed that supportive equipment was used in studies analysing competition data.”*

*We have added the following information to the results (lines 252 to 254):*

*“Supportive equipment was used during 1RM assessments in 11 studies, including four studies involving Para-athletes and seven studies involving non-disabled athletes.”*

5) Table 2 – waist circumference is listed twice (under upper limb and trunk categories) with slightly different correlation coefficients reported from the same study (reference #6). Please clarify.

Response: *Thank you for this comment. Table 2 contains* ***wrist*** *circumference and* ***waist*** *circumference. Both measures are from reference number 6 (Ferrari et al. 2022). We have checked the reference and confirm the correlation coefficients for both measures are correct. The table from Ferrari and colleagues (2022) including the correlation coefficients for wrist and waist circumference is pasted below.*

6) Figure 4 is not brought up in the manuscript until the discussion. In my opinion, a description of this figure should first be mentioned in the results section, with commentary on the implications of this in the discussion.

Response: *We have referred to Figure 4 in the data analysis section of the results. We have added discussion of implications of failing to consider uncertainty around correlation coefficients on practice (lines 482 to 486):*

*“Failure to consider uncertainty of correlations may encourage performance staff to adopt weak evidence in their practice. For example, several trainable anthropometric factors have moderate correlation coefficients but their confidence intervals cross zero (see Figure 4). A focus on these factors may waste time that could be better spent focusing on other trainable factors underpinning performance.”*

7) The discussion proposes sound recommendations for future research based on the previous studies reviewed.

Response: *We thank the reviewer for their positive comments.*

**Reviewer #3**

Response: *We would like to thank the reviewer for their time in providing comment on the manuscript. Addressing the concerns raised has improved the quality of the manuscript.*

General comments:
Title Are presented satisfactorily.

Response: *Thank you.*

Abstract
It is written in a structured way, however, the methodology is written in a very summarized way which ends up making the findings and conclusions of the article.

Response: *We have added to the abstract based on comments from the reviewer below. We hope this satisfies any concerns surrounding the abstract of the study. We have pasted the current abstract below, with updated sections highlighted:*

*“Purpose: Understanding important features for performance in non-disabled bench press and Paralympic powerlifting may inform talent identification and transfer models. The aim of this scoping review was to describe features associated with bench press performance.*

*Methods: We conducted a systematic search of three electronic databases (PubMed, SportDiscus and EMBASE) to identify studies involving non-disabled and Para athlete populations that investigated features related to bench press one-repetition maximum (1RM), across six domains (anthropometric, body composition, demographic, technical, disability and neuromuscular). Search terms included “resistance training”, “para powerlifting’ and “one repetition max”. No date restrictions were include in searches. Studies using adult participants, with at least six-months of bench press experience, who were able to bench press their body mass were included.*

*Results: Thirty-two studies met our inclusion criteria. Twenty-four studies involved non-disabled athletes (total n = 2,686; 21.9% female) and eight involved Para athletes (total n = 2,364; 39.4% female). Anthropometric (17 studies) and body composition (12 studies) features were most studied; half of the 32 studies investigated features from a single domain. Of anthropometric variables, upper arm circumference shared the strongest association with bench press 1RM in non-disabled (r = 0.87) and para-athletes (r = 0.65). Upper limb fat free mass (r = 0.91) and body mass index (r = 0.46) were the body composition variables sharing the strongest association with bench press 1RM in non-disabled and para-athletes. Few studies considering the uncertainty of their results. Practices of open and transparent science, such as pre-registration and data sharing, were absent.*

*Conclusion: The development of bench press talent identification and sport transfer models will require future studies to investigate both non-training and training modifiable features, across multiple domains. Large longitudinal studies using information from athlete monitoring databases and multivariable model approaches are needed to understand the interacting features associated with bench press performance, and for the development of talent identification models.*

It would be very important to have more details on how the study was carried out, such as query words, length of time it was consulted, that is, articles published within a certain period of time.

Response: *We have stated that no date restrictions were put on searches and have added key words to the abstract as requested.*

Furthermore, more numerical data should be presented in order to provide an initial overview of the manuscript.

Response: *We have added numerical data to the results in the abstract, including specific variables sharing the strongest association with bench press 1RM (and their correlation coefficient), as requested.*

*“Results: Thirty-two studies met our inclusion criteria. Twenty-four studies involved non-disabled athletes (total n = 2,686; 21.9% female) and eight involved Para athletes (total n = 2,364; 39.4% female). Anthropometric (17 studies) and body composition (12 studies) features were most studied; half of the 32 studies investigated features from a single domain. Of anthropometric variables, upper arm circumference shared the strongest association with bench press 1RM in non-disabled (r = 0.87) and para-athletes (r = 0.65). Upper limb fat free mass (r = 0.91) and body mass index (r = 0.46) were the body composition variables sharing the strongest association with bench press 1RM in non-disabled and para-athletes. There was a primary reliance on bivariate correlation analysis (56% of studies), with Few studies considering the uncertainty of their results. Practices of open and transparent science, such as pre-registration and data sharing, were absent.”*

Please confirm that the keywords are listed as descriptors in health sciences.

Response: *Keywords have been altered to ensure they are MeSH terms in PubMed.*

Introduction

Although the introduction is of a very good length, and initially with a contextualization of Powerlifting, whether conventional or palympic, and more specifically the bench press. However, the introduction cannot move from the general to the specific, that is, the association between performance and anthropometric characteristics associated with health. And on the other hand, the problem is not clear, not allowing us to understand the objectives, much less justify them. It would also be important to present research hypotheses to be answered.

Response: *We apologize for any lack of clarity in the introduction. We have made some minor amendments to help the reader better understand the problem the study aims to address and the study objectives (lines 83 to 85 & 89 to 91):*

*“Many sports use talent identification models encompassing a range of anthropometric, physical performance and cognitive variables ^17^ but no uniform approach is currently available for in powerlifting.”*

*“Para athletes often report difficulty transitioning into retirement ^19^ and prolonging their career by transferring into a sport with an older age of peak performance may be beneficial for para athlete well-being.”*

*While we want to satisfy as many of the reviewers concerns as possible, we are not comfortable generating hypotheses after conducting the study. We have pre-registered the study and have not declared any hypotheses in the pre-registration (we are also not conducting any null hypothesis significance testing). Hypothesizing after the results are known has been identified as a questionable research practice (Caldwell et al. 2020. Sports Medicine) and we hope the reviewer understands that we are not comfortable adding hypotheses to the introduction after conducting the study. Additionally, the aim of a scoping review is to summarize and describe findings within a research area (Arksey and O’Malley. 2005. International Journal of Social Research Methodology) and hypothesizing is typically not consistent with the aims of a scoping review.*

Methods

It should present more clearly the design of the study.

Response: *Thank you. As per the scoping review guidelines, we have stated the study design (i.e. a scoping review) in the title and in the first sentence of the methods.*

The keywords researcher and the period of time researched are not included in the manuscript. As it turns out, the database and the language, therefore, the methodology is not complete, it would be good to complement it.

Response: *We apologize for this oversight. We have added examples of key words used in search strategies, and that our search strategy did not include date restrictions to the methods. Given search strategies are long and complicated, we have included the specific search strategies used in each database as a supplement (Supplement 1). Our preference was to provide the specific strategies, such that the searches could be replicated, in full.*

Results
Are presented satisfactorily.

Response: *We thank the reviewer for their positive feedback.*

Discussion
It should reaffirm the objectives and start discussing the results in the chronological order that appear in the item results.

Response: *We have restructured the discussion to begin with a summary of the results in relation to the aims of the study, followed by population characteristics, then a discussion of important features of bench press performance and finally a discussion of data analyses in included studies. This is consistent with the order of presentation in the results, as requested by the reviewer. We hope this amendment satisfies this concern.*

Please present the limitations of the study.

Response: *We have included a section titled “Limitations” after the discussion. The limitations section reads:*

*“Several limitations of the review require acknowledgment. First, consistent with scoping review practices, we did not consider the quality of the 32 studies in the review. Second, we did not formally aggregate correlation coefficients using meta-analytical modelling. Our intention was to summarise the features investigated, rather than comment specifically on the strength of associations between certain features and bench press 1RM. Nonetheless, several features that were investigated by multiple studies, such as chest circumference (Table 2), fat mass (Table 3) and age (Table 4), were deemed to have an unclear association with bench press 1RM. It is possible that formal aggregation of these studies by conducting a meta-analysis would show a positive association with bench press 1RM. Third, our review underestimates the number of females included in studies of non-disabled athletes, as female groups from three studies ^7,15,31^ were excluded after applying our inclusion criteria. Including the female groups from these three studies would have changed the proportion of females studied from 21.9% to 34.9%; however, it would not have changed our substantive conclusion that female athletes were generally underrepresented across the 32 studies in the review.*

Conclusion
Are presented satisfactorily.

Response: *We thank the reviewer for their positive feedback.*

References

Are presented satisfactorily, review their formatting. Of the 71 references, 37 are current and 34 have been published for more than five years. In this sense, it would be good to check current studies and, if necessary, include them in the manuscript.

Response: *As requested, we have checked references published in the last five years. All of these references are either included in the review, or, did not meet our inclusion criteria.*

Overview
The manuscript presented addresses a relevant research topic.
It would be advisable to do a general review.

Response: *We thank the reviewer for their positive feedback. We hope we have addressed any concerns in responses to the above comments. We have also included a general overview, as requested.*
